# A clinical-radiomics nomogram based on multimodal ultrasound for predicting the malignancy risk in solid hypoechoic breast lesions

**DOI:** 10.3389/fonc.2023.1256146

**Published:** 2023-10-17

**Authors:** Guo Shiyan, Jiang Liqing, Yan Yueqiong, Zhang Yan

**Affiliations:** Department of Ultrasound, Third Xiangya Hospital, Central South University, Changsha, Hunan, China

**Keywords:** nomogram, breast, radiomics, automated breast volume scanner, strain elastography

## Abstract

**Background:**

In routine clinical examinations, solid hypoechoic breast lesions are frequently encountered, but accurately distinguishing them poses a challenge. This study proposed a clinical-radiomics nomogram based on multimodal ultrasound that enhances the diagnostic accuracy for solid hypoechoic breast lesions.

**Method:**

This retrospective study analyzed ultrasound strain elastography (SE) and automated breast volume scanner images (ABVS) of 423 solid hypoechoic breast lesions from 423 female patients in our hospital between August 2019 and May 2022. They were assigned to the training (n=296) and validation (n=127) groups in a 7:3 ratio by generating random numbers. Radiomics features were extracted and screened from ABVS and SE images, followed by the calculation of the radiomics score (Radscore) based on these features. Subsequently, a nomogram was constructed through multivariate logistic regression to assess the malignancy risk in breast lesions by combining Radscore with Breast Imaging Reporting and Data System (BI-RADS) scores and clinical risk factors associated with breast malignant lesions. The diagnostic performance, calibration performance, and clinical usefulness of the nomogram were assessed by the area under the curve (AUC) of the receiver operating characteristic curve, the calibration curve, and the decision analysis curve, respectively.

**Results:**

The diagnostic performance of the nomogram is significantly superior to that of both the clinical diagnostic model (BI-RADS model) and the multimodal radiomics model (SE+ABVS radiomics model) in training (AUC: 0.972 vs 0.930 vs 0.941) and validation group (AUC:0.964 vs 0.916 vs 0.933). In addition, the nomogram also exhibited a favorable goodness-of-fit and could lead to greater net benefits for patients.

**Conclusion:**

The nomogram enables a more effective assessment of the malignancy risk of solid hypoechoic breast lesions; therefore, it can serve as a new and efficient diagnostic tool for clinical diagnosis.

## 1 Introduction

As the most prevalent cancer in the world, breast cancer poses a grave threat to people’s health and survival (1). Given its high metastatic tendency and high mortality rate (2, 3), coupled with the significant differences in treatment modalities for benign and malignant breast tumors, early definitive diagnosis is a critical first step in the therapeutic management of breast lesions, which plays a crucial role in improving patient outcomes and survival (3–5).

With the recent advancements in ultrasound imaging technology, ultrasound plays an increasingly important role in the detection of breast lesions. Strain elastography (SE) allows for a quick and intuitive display of differences in elasticity coefficients within the lesion through color-coded imaging, therefore, it serves as a powerful diagnostic aid to offer valuable reference values for lesion diagnosis (6, 7). Automated breast volume scanner (ABVS) provides good reproducibility of diagnostic results due to its standardized operating procedures (8), it can acquire the whole breast volume information and perform multiplanar imaging on the acquired information. Studies have shown that ABVS exhibits comparable diagnostic accuracy to handheld ultrasound scanners for detecting breast lesions, while also providing additional information (9, 10). In routine ultrasound examinations, it is frequent to encounter patients with solid hypoechoic breast lesions, physicians can make an initial assessment of the malignancy risk of breast lesions based on their morphological appearance on ABVS image and elastic performance on SE images. The combination of ABVS and SE imaging techniques demonstrates significant diagnostic efficacy in evaluating breast lesions (11). However, the dependability of diagnostic outcomes generated by conventional imaging techniques is largely contingent on the proficiency of the examining physician and is markedly susceptible to interobserver variability (12).

Radiomics is in line with the current trend toward precision medicine, as it transforms ordinary visual images into high-throughput data through deep mining of medical images, allowing for the capturing of the internal heterogeneity of the entire tumor in a non-invasive manner (13–15). Therefore, it may provide novel biomarkers to facilitate diagnosis for better clinical decision-making. There are already several radiomics studies on ultrasound (US), mammography, and magnetic resonance (MR) in breast cancer diagnosis that have yielded promising results (16–29). However, there have been no studies on the combination of ABVS and UE radiomics features with clinical ultrasound factors for the diagnosis of breast cancer. Therefore, we conducted a radiomics analysis on SE and ABVS images, then combined these features with traditional imaging risk assessments and other clinical risk factors, resulting in a novel nomogram to help physicians accurately diagnose solid hypoechoic breast lesions.

## 2 Materials and methods

### 2.1 Patients

**Figure 1:**
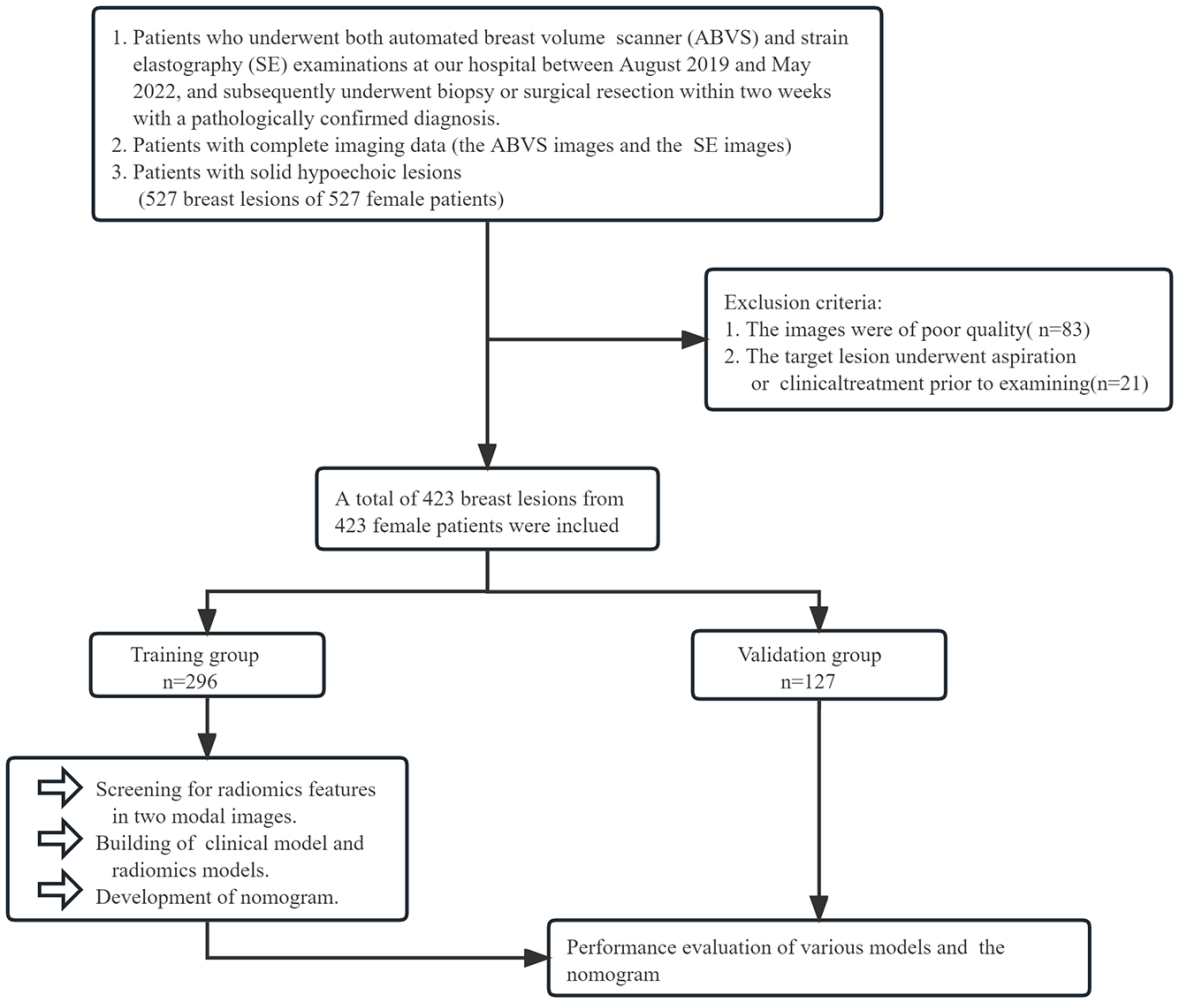
The grouping process of this study.

The retrospective study was approved by the institutional review board at our hospital. The inclusion criteria were: (1) Patients who underwent both ABVS and SE examinations at our hospital between August 2019 and May 2022 and subsequently underwent biopsy or surgical resection within two weeks with a pathologically confirmed diagnosis. (2) Patients with complete imaging data. (3) Patients’ breast lesions were hypoechoic solid lesions. The exclusion criteria were: (1) Patients whose images were of poor quality; (2) Patients who underwent aspiration or clinical treatment before examining target lesions. Eventually, we included 423 solid hypoechoic breast lesions from 423 female patients. By generating random numbers, they were allocated into training and validation groups in the ratio of 7:3. The flow is shown in **Figure 1**.

### 2.2 Image acquisition and assessment

In this study, all images used were obtained using the ACUSON S2000 US machine and its accompanying ABVS system.

#### 2.2.1 SE image

Patients were instructed to breathe normally while lying supine on the examination bed with their breasts fully exposed. A 9L4 probe in two-dimensional ultrasound mode was used to examine the breast in all planes. The imaging mode was then switched to the elastic mode when scanning the largest two-dimensional section of the lesions, and the patient was required to cooperate by holding her breath. The probe is placed perpendicularly over the breast without applying any pressure during the capture of SE images, with the lesion positioned at the center of an elasticity sampling window at least twice the size of the area of interest.

#### 2.2.2 ABVS image

Instruct the patient to raise both arms over the head and remain in the supine position. A sufficient amount of coupling agent was applied uniformly to the breast. Before scanning, parameters such as depth and overall gain were adjusted to achieve optimal image quality. During the scanning procedure, patients were instructed to breathe normally. Each breast was routinely scanned in two positions using a 14L5BV high-frequency linear array automatic scanning US probe. The nipples were marked after the scanning, and the acquired images were saved and transferred to a workstation for processing and analysis. Images with the maximum section image of the target lesion in coronal, transverse, and sagittal planes were selected for subsequent region of interest (ROI) segmentation and feature extraction.

Refer to the BI-RADS criteria which were defined by the American College of Radiology in 2013 (30), we evaluated the morphology, margin, border, orientation, posterior echogenicity of the lesion, microcalcification within the lesion, and conditions of retraction in its coronal plane on the saved images. This is followed by a combination with ultrasound elastic strain performance of the lesions (elasticity score using a 5-point scale (31)), enabling an accurate classification of the risk of malignancy of the lesions.

All of the above steps were performed by a physician who has over ten years of expertise in ultrasound breast disease diagnosis.

### 2.3 Extraction and selection of radiomics features

**Figure 2:**
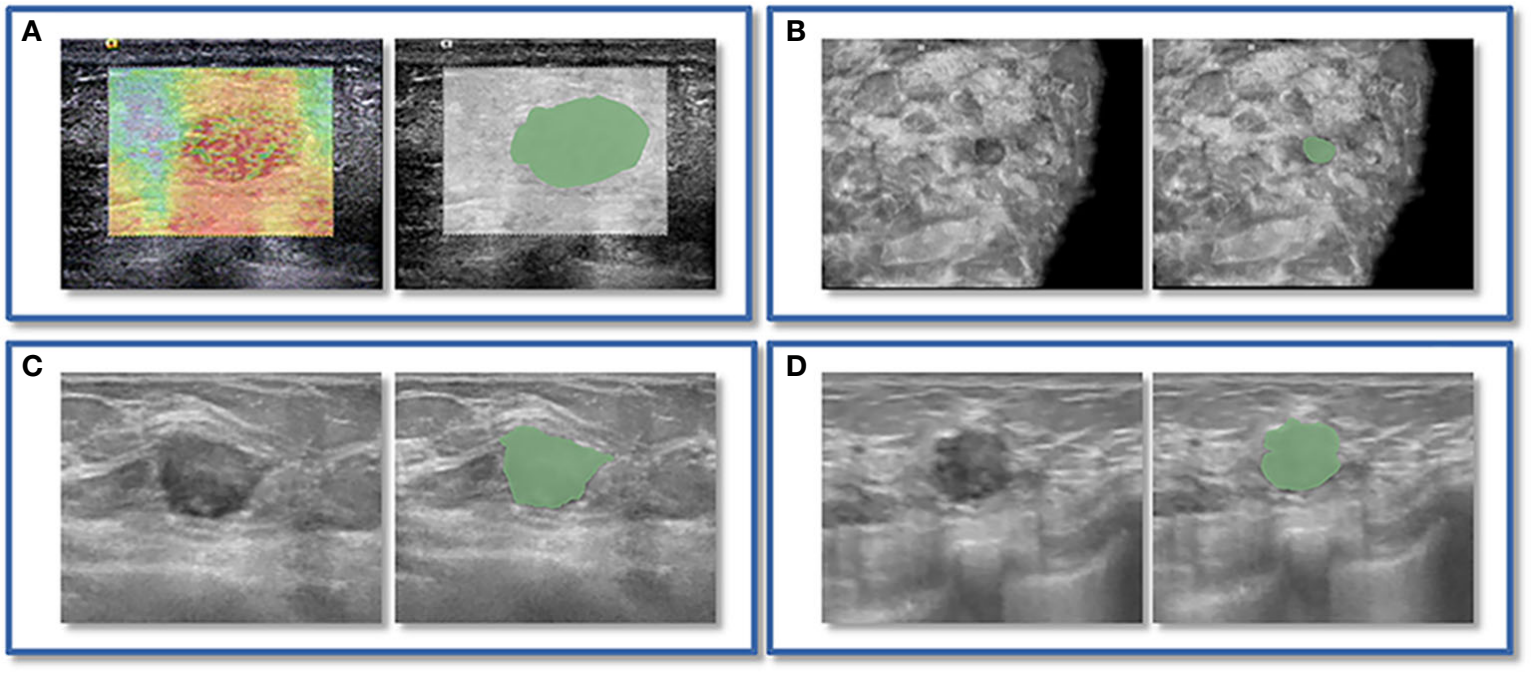
An instance of manually delineating a region of interest (ROI). The strain elastography (SE) and automated breast volume scanner (ABVS) images of a 41-year-old female with a solid hypoechoic lesion measuring approximately 16x11x12mm on her left breast. The lesion was irregular in shape, parallel in position, with still well-defined borders, sharp margins, and scattered microcalcifications visible internally, and exhibited no significant posterior echogenicity change or retraction in the coronal plane, and the ultrasound elasticity score was 4, finally, the lesion was classified as BI-RADS category 4a. Pathological examination confirmed it as invasive ductal carcinoma. ROI segmentation was performed on both the SE image **(A)** and ABVS coronal image **(B)**, with delineation along the boundary of the lesion followed by uniform outward expansion of its edges by 3 mm to encompass some surrounding tissue.ROI segmentation was performed on ABVS transverse **(C)** and sagittal **(D)** images, respectively, and meticulous delineation was performed along the lesion’s contour and borders on these two views.

The ABVS and SE images were sequentially imported into 3D Slicer 5.2.1 for image processing, manual segmentation of the ROI and extraction of radiomics features. We delineated ROI for lesions in both SE and ABVS images. Notably, in the ABVS images, we delineated the ROI on the coronal, sagittal, and transverse planes of the lesions, respectively. Further details can be found in **Figure 2**. This procedure was performed with the participation of two physicians. Physician A, with five years of experience in ultrasound-based breast disease diagnosis, performed outlining for all the lesions. Physician B, with eight years of experience in ultrasound-based breast disease diagnosis, conducted lesion outlining on the training group to validate ROI outlining reproducibility. Then, we utilized the Pyradiomics package within 3D Slicer to extract radiomics features from the SE and ABVS images, respectively. The features extracted encompassed first-order statistics features, texture features (including the gray level co-occurrence matrix (glcm), gray level dependence matrix (gldm), gray level run length matrix (glrlm), gray level size zone matrix (glszm), and the neighbouring gray tone difference matrix (ngtdm)), as well as post-wavelet transformed features. Subsequently, we subjected the extracted features to screening. By utilizing the intra-class correlation coefficient (ICC) analysis, we can identify features that exhibit high levels of reproducibility (ICC > 0.75) (32), subsequently, radiomics features extracted from the region of interest segmented by physician A were utilized for further analysis. All feature values were normalized using Zscore. The radiomics features of both modalities were subjected to dimensionality reduction through the Mann-Whitney U test and least absolute shrinkage and selection operator (LASSO) regression and to identify features with strong qualitative diagnostic ability for solid hypoechoic breast lesions.

### 2.4 Development of models

#### 2.4.1 Radiomics models

Based on radiomics features of SE and ABVS images, logistic regression analysis was utilized to build the radiomics model, including the SE radiomics model, the ABVS radiomics model, the SE+ABVS radiomics model that was developed by combining the two. The radiomics score (Radscore) for each lesion was computed by weighting the coefficients of features in the SE+ABVS radiomics model.

#### 2.4.2 Clinical diagnostic model

The BI-RADS model was constructed through logistic regression analysis of lesion’s BI-RADS categories in the training group.

### 2.5 Development and performance validation of nomogram

This study performed a univariate analysis in order to determine the risk predictive variables associated with breast cancer(*P*<0.05), which were then combined with the results of conventional imaging assessment and radiomics analysis. Based on these findings, we integrated relevant clinical risk factors, the Radscore, and the BI-RADS category of lesions to develop a nomogram for assessing the malignancy risk in such breast lesions by multivariate logistic regression analysis. Subsequently, the nomogram’s diagnostic performance was compared to that of the BI-RADS model and SE+ABVS radiomics model. To evaluate the diagnostic performance of the models, we calculated the area under the receiver operating characteristic curve (AUC) for each model in the training group, validation group, and in the BI-RADS category 4 lesions within both groups. Furthermore, the DeLong test was used to examine differences in AUC values between different models. The nomogram’s goodness of fit was investigated graphically and by calculating significance by plotting the calibration curve and conducting the Hosmer-Lemeshow test. Lastly, clinical decision analysis curves were drawn for quantifying the net benefits of the BI-RADS model, SE+ABVS radiomics models, and nomogram at various threshold probabilities.

### 2.6 Statistical analysis

SPSS 23.0, R 4.2.2, and MedCalc 19.6.0 were utilized for statistical analysis and graph plotting. The ‘psych’, ‘survival’, ‘glmnet’, ‘rms’, ‘ResourceSelection’, and ‘rmda’ packages were used in R. We performed normality tests on each group of data and selected the appropriate hypothesis test based on the results to compare the distribution of data between the training and validation groups. The study has chosen a significance level of 0.05 as the threshold for detecting statistical differences.

## 3 Results

### 3.1 Comparison of clinical basis information and sonographic features

The study included 423 breast lesions that were pathologically confirmed to include 215 benign lesions and 208 malignant lesions. **Table 1** demonstrates that both the clinical basis data and sonographic features of lesions were evenly distributed in the training and validation groups, indicating no statistically significant differences between the two groups (*P*>0.05). Furthermore, the univariate risk analysis revealed that patients with malignant lesions had significantly higher age and lesion’s maximum diameter compared to those with benign lesions in both groups (*P*<0.05). However, no correlation was seen between the location of the lesion and the malignant risk of the lesion (*P*>0.05). Hence, we regarded age and lesion size as predictor variables in the context of breast cancer. Regarding the sonographic features of the lesions, there were statistically significant differences (*P*<0.01) observed in morphology, borders, margins, orientation, microcalcifications, retraction condition of the coronal plane, and elasticity scores between benign and malignant lesions within both groups. while no statistical differences were found in posterior echogenicity (*P*>0.05). This study assessed the malignancy risk of lesions by these sonographic features of them, and the BI-RADS categories obtained were also significantly different in benign and malignant lesions (*P*<0.01).

**Table 1:** Clinical basis information and sonographic features of patients with breast lesions.

Characteristic	Training group (n=296)			Validation group (n=127)			
	Benign group (n=151)	Malignant group (n=145)	*P*_Intra_ value	Benign group (n=64)	Malignant group (n=63)	*P*_Intra_ *value*	*P*_Inter_ *value*
**Age**							
Median±SD	39±10.85	51±10.73	<0.01*	41±9.83	53±10.82	<0.01*	0.34
**maximum diameter**							
Median±SD	13±7.36	19±8.23	<0.01*	15±9.24	18±8.52	<0.05*	0.37
**Location**							
Left	72 (47.68%)	79 (54.48%)	0.24	32 (50%)	30 (47.62%)	0.79	0.68
right	79 (52.32%)	66 (45.52%)		32 (50%)	33 (52.39%)		
**Morphology**							
Regular	77 (50.99%)	17 (11.72%)	<0.01*	28 (43.75%)	5 (7.94%)	<0.01*	0.24
Irregular	74 (49.01%)	128 (88.28%)		36 (56.25%)	58 (92.06%)		
**Border**							
Clear	110 (72.85%)	45 (31.03%)	<0.01*	43 (67.19%)	19 (30.16%)	<0.01*	0.50
Not Clear	41 (27.15%)	100 (68.94%)		21 (32.81%)	44 (69.84%)		
**Margin**							
Circumscribed	127 (84.11%)	22 (15.17%)	<0.01*	51 (79.69%)	12 (19.05%)	<0.01*	0.89
Not circumscribed	24 (15.89%)	123 (84.83%)		13 (20.31%)	51 (80.95%)		
**Orientation**							
Parallel	128 (84.77%)	79 (54.48%)	<0.01*	54 (84.38%)	32 (50.79%)	<0.01*	0.65
Not parallel	23 (15.23%)	66 (45.52%)		10 (15.62%)	31 (49.21%)		
**Posterior echogenicity**							
Enhancement	11 (7.28%)	17 (11.72%)	0.28	4 (6.25%)	6 (9.52%)	0.82	0.52
No difference	130 (86.10%)	111 (76.56%)		57 (89.06%)	52 (82.54%)		
Shadowing	10 (6.62%)	17 (11.72%)		3 (4.69%)	5 (7.94%)		
**Retraction sign**							
Presence	0 (0%)	32 (22.07%)	<0.01*	0 (0%)	16 (25.40%)	<0.01*	0.60
Absence	151 (100%)	113 (77.93%)		64 (100%)	47 (74.60%)		
**MicroCalcification**							
Presence	16 (10.60%)	66 (45.52%)	<0.01*	3 (4.69%)	26 (41.27%)	<0.01*	0.30
Absence	135 (89.40%)	79 (54.48%)		61 (95.31%)	37 (58.73%)		
**Ultrasonic elasticity score**							
1 point	16 (10.60%)	0 (0%)		7 (10.94%)	0 (0%)		
2 points	49 (32.45%)	1 (0.69%)		22 (34.37%)	3 (4.76%)		
3 points	65 (43.05%)	21 (14.48%)	<0.01*	27 (42.19%)	14 (22.22%)	<0.01*	0.70
4 points	20 (13.24%)	55 (37.93%)		8 (12.50%)	24 (38.10%)		
5 points	1 (0.66%)	68 (46.90%)		0 (0%)	22 (34.92%)		
**BI-RADS**							
3	64 (42.38%)	4 (2.76%)		27 (42.19%)	2 (3.17%)		
4a	82 (54.31%)	21 (14.48%)	<0.01*	34 (53.12%)	11 (17.46%)	<0.01*	0.77
4b	4 (2.65%)	27 (18.62%)		3 (4.69%)	12 (19.05%)		
4c	1 (0.66%)	30 (20.69%)		0 (0%)	17 (26.98%)		
5	0 (0%)	63 (43.45%)		0 (0%)	21 (33.34%)		

* *p* < 0.05.

### 3.2.Screening of radiomics features

**Figure 3:**
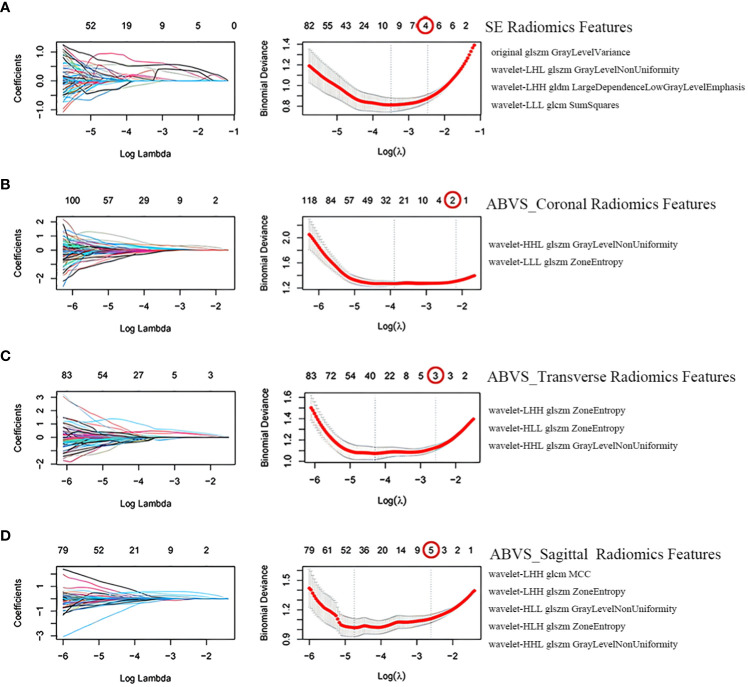
Screening of radiomics features. Selection of strain elastography (SE) radiomics features **(A)**, automated breast volume scanner (ABVS) coronal plane radiomics features **(B)**, ABVS transverse plane radiomics features **(C)**, and ABVS sagittal plane radiomics features **(D)** using the least absolute shrinkage and selection operator (LASSO) regression model. The coefficient profiles of LASSO for each modal radiomics feature are presented on the left. The right shows that the tuning parameter λ (lambda) in the LASSO model was selected using tenfold cross-validation, and the binomial deviance was plotted as a function of log(λ), with vertical dashed lines drawn at the minimum deviation (log(λ.min)) and the 1 standard error of the minimum deviation (log(λ.1se)). Selected the non-zero coefficient features in the model when the horizontal coordinate was log(λ.1se).

The SE and ABVS images generated 837 and 2511 radiomics features (ABVS cross plane, sagittal plane, and coronal plane, each generated 837 features), respectively. The training group’s SE radiomics features as well as the ABVS coronal plane, transverse plane, and sagittal plane radiomics features underwent sequential ICC analysis, Mann-Whitney U test, LASSO regression analysis with tenfold cross-validation for dimensionality reduction. Finally, a total of 14 features were selected, comprising four SE radiomics features and ten ABVS radiomics features (two from the coronal plane, three from the transverse plane, and five from the sagittal plane). All of these radiomics features are texture features, one of which was from the original image and thirteen were obtained after wavelet transform (**Figure 3**).

### 3.3 Comparison of radiomics models

**Figure 4:**
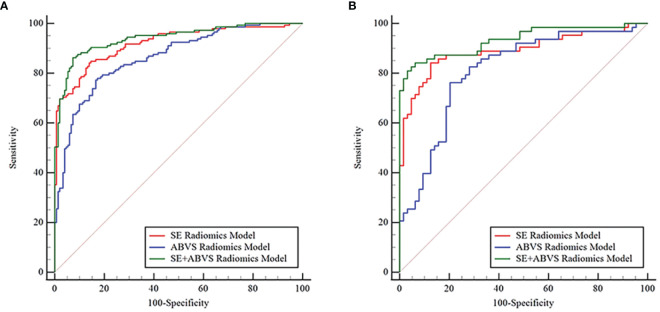
The receiver operator characteristic curves for various radiomics models in the training **(A)** and validation groups **(B)**.

By comparing and validating the diagnostic efficacy of the radiomics models (**Figure 4**, **Table 2**), the AUC values of the selected ABVS and SE features for distinguishing between benign and malignant solid hypoechoic breast lesions were consistently above 0.8 in both the training and validation groups. Moreover, compared to any single-modality radiomics models, the SE+ABVS radiomics model, which integrated the radiomics features of two imaging modalities, demonstrated significantly higher AUC values in both training (All *P*<0.01) and validation groups (compared to the ABVS radiomics model: *P*<0.01, compared to SE radiomics model: *P*<0.05). These outcomes suggest that combining radiomics features from both SE and ABVS could enhance the accuracy of diagnostic models. Thus, the Radscore for each patient was obtained by weighting the corresponding coefficients for each feature in the SE+ABVS radiomics model., the formula is shown below, the Radscore for malignant lesions was found to be significantly higher than that for benign lesions within both groups. (Training group: 2.86 + 2.66, -2.34 + 1.80, *P*<0.01; Validation group: 2.60 + 2.31, -2.17 + 1.85, *P*<0.01).

**Table 2:** The AUC values of radiomics models in the training and validation groups.

Model	AUC (95%CI)	*P* (AUC compare to SE radiomics model)	*P* (AUC compare to ABVS radiomics model)
Training group			
SE radiomics model	0.920 (0.883, 0.948)		<0.01*
ABVS radiomics model	0.865 (0.821, 0.902)	<0.01*	
SE+ABVS radiomics model	0.941 (0.907, 0.965)	<0.01*	<0.01*
Validation group			
SE radiomics model	0.892 (0.824, 0.940)		0.08
ABVS radiomics model	0.811 (0.732,0.875)	0.08	
SE+ABVS radiomics model	0.933 (0.875,0.970)	<0.05*	<0.01*

*P< 0.05.


$$\begin{array}{l} \text{Radscore}=1.944469*\text{original glszm GrayLevelVariance}\_\text{SE}\\ +0.03492*\text{wavelet}-L\text{HL glszm GrayLevelNonUniformity}\_\text{SE}\\ +0.634491*\text{wavelet}-\text{LHH gldm LargeDependenceLowGrayLevelEmphasis}\_\text{SE}\\ +0.465968*\text{wavelet}-\text{LLL glcm SumSquares}\_\text{SE}\\ +0.133391*\text{wavelet}-\text{HHL glszm GrayLevelNonUniformity}\_\text{ABVS}\_\text{Coronal}\\ -1.515639*\text{wavelet}-\text{LLL glszm ZoneEntropy}\_\text{ABVS}\_\text{Coronal}\\ +0.010666*\text{wavelet}-\text{LHH glszm ZoneEntropy}\_\text{ ABVS}\_\text{Transverse}\\ +1.292836*\text{wavelet}-\text{HLL glszm ZoneEntropy}\_\text{ ABVS}\_\text{Transverse}\\ +0.043941*\text{wavelet}-\text{HHL glszm GrayLevelNonUniformity}\_\text{ ABVS}\_\text{Transverse}\\ -1.828919*\text{wavelet}-\text{LHH glcm MCC}\_\text{ABVS}\_\text{Sagittal}\\ +0.813141*\text{wavelet}-\text{LHH glszm ZoneEntropy}\_\text{ABVS}\_\text{Sagittal}\\ -0.008279*\text{wavelet}-\text{HLL glszm GrayLevelNonUniformity}\_\text{ABVS}\_\text{Sagittal}\\ +1.520859*\text{wavelet}-\text{HLH glszm ZoneEntropy}\_\text{ABVS}\_\text{Sagittal}\\ +0.271946*\text{wavelet}-\text{HHL glszm GrayLevelNonUniformity}\_\text{ABVS}\_\text{Sagittal}\\ -25.66532 \end{array}$$

### 3.4 Evaluation of nomogram performance

**Figure 5:**
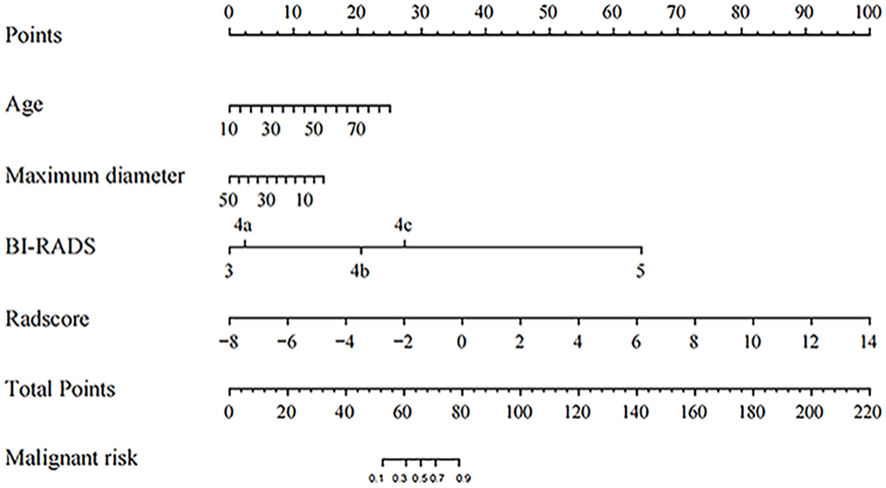
The Nomogram for predicting the malignant risk of solid hypoechoic breast lesions.

Based on the clinical risk factors identified through univariate analysis, BI-RADS categories determined from imaging assessments, and Radscore obtained from radiomics analysis, we constructed a nomogram using multivariate logistic regression to visually assess the risk of malignancy in solid hypoechoic breast lesions, the nomogram incorporated the patient’s age, lesion’s maximum diameter, Radscore, and BI-RADS category. As illustrated in **Figure 5**, Radscore had the highest weightage followed by BI-RADS score while age and maximum diameter of the lesion exerted less influence on assessment results.

**Figure 6:**
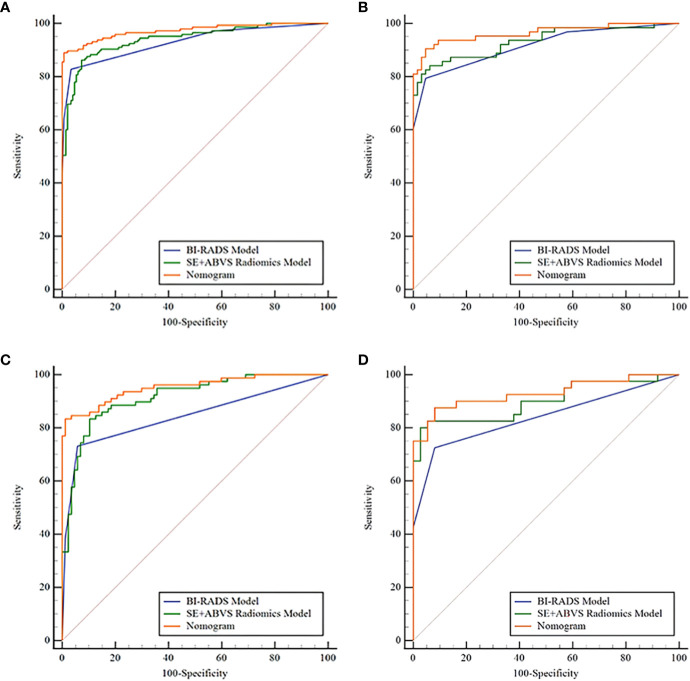
The receiver operator characteristic curves of the BI-RADS model, SE+ABVS radiomics model, and Nomogram in the training group **(A)**, the validation group **(B)**, the BI-RADS category 4 lesions in the training group **(C)**, and the BI-RADS category 4 lesions in the validation group **(D)**.

**Figure 6** and **Table 3** present that the BI-RADS model, SE+ABVS radiomics model, and nomogram are effective in predicting the malignancy risk in solid hypoechoic breast lesions, Notably, the nomogram exhibits superior diagnostic performance with higher AUC values (0.972, 0.964) in training and validation group compared to both the BI-RADS model (AUC: 0.930, 0.916) and SE+ABVS radiomics models (AUC: 0.941, 0.933). Furthermore, its difference with BI-RADS model and SE+ABVS radiomics model was statistically significant in both groups (*P*<0.05). Besides, we further compared the diagnostic efficacy of the three models for BI-RADS category 4 lesions within the two groups. The results revealed that the nomogram (AUC: 0.952, 0.930) consistently exhibited higher AUC values than both the BI-RADS model (AUC:0.844, 0.839) and SE+ABVS radiomics model (AUC:0.915, 0.899). Moreover, there were consistently statistically significant differences between the nomogram and BI-RADS model (All *P*<0.01). However, in comparison to the SE+ABVS model, the nomogram was only statistically different from it in the training group (*P*<0.05), but not in the validation group (*P*>0.05). Other than that, in terms of diagnostic sensitivity, specificity, and accuracy, Although the specificity of the nomogram was slightly inferior to that of the BI-RADS model in the training group, it significantly improved diagnostic sensitivity. Furthermore, its diagnostic parameters were at the highest level across all validation groups. These results suggest that the nomogram exhibited the best overall diagnostic performance. Finally, we observed that the AUC values of the SE+ABVS Radiomics model consistently outperformed those of the BI-RADS model, and a statistically significant difference was found between them when diagnosing BI-RADS category 4 lesions of the training group *(P*<0.05). This finding highlights the ability of radiomics analysis to detect deep-seated features within the images, ultimately leading to improved diagnostic efficiency.

**Table 3:** The diagnostic parameters of the BI-RADS model, SE+ABVS radiomics model, and Nomogram in each group.

Model	AUC (95%CI)	Sensitivity%	Specificity%	Accuracy%	*P* (AUC compare to BI-RADS model)	*P* (AUC compare to SE+ABVS radiomics model)
Training group						
BI-RADS model	0.930(0.894, 0.956)	82.76	96.69	89.86		0.46
SE+ABVS radiomics model	0.941(0.907, 0.965)	87.59	90.07	88.85	0.46.	
Nomogram	0.972(0.946, 0.988)	89.66	94.04	91.89	<0.01*	<0.01*
Validation group						
BI-RADS model	0.916(0.853, 0.958)	79.37	95.31	87.40		0.52
SE+ABVS radiomics model	0.933(0.875, 0.970)	85.71	85.94	85.83	0.52	
Nomogram	0.964(0.916, 0.989)	87.30	95.31	91.34	<0.05*	<0.05*
BI-RADS category 4 lesions in the Training group						
BI-RADS model	0.844(0.779, 0.895)	73.08	94.25	84.24		<0.05*
SE+ABVS radiomics model	0.915(0.862, 0.953)	84.62	87.36	86.06	<0.05*	
Nomogram	0.952(0.907, 0.979)	84.62	89.66	87.27	<0.01*	<0.05*
BI-RADS category 4 lesions in the Validation group						
BI-RADS model	0.839(0.738, 0.913)	72.50	91.89	81.82		0.13
SE+ABVS radiomics model	0.899(0.809, 0.956)	82.50	83.78	83.12	0.13	
Nomogram	0.930(0.848, 0.975)	82.50	91.89	87.01	<0.01*	0.12

*P<0.05.

**Figure 7:**
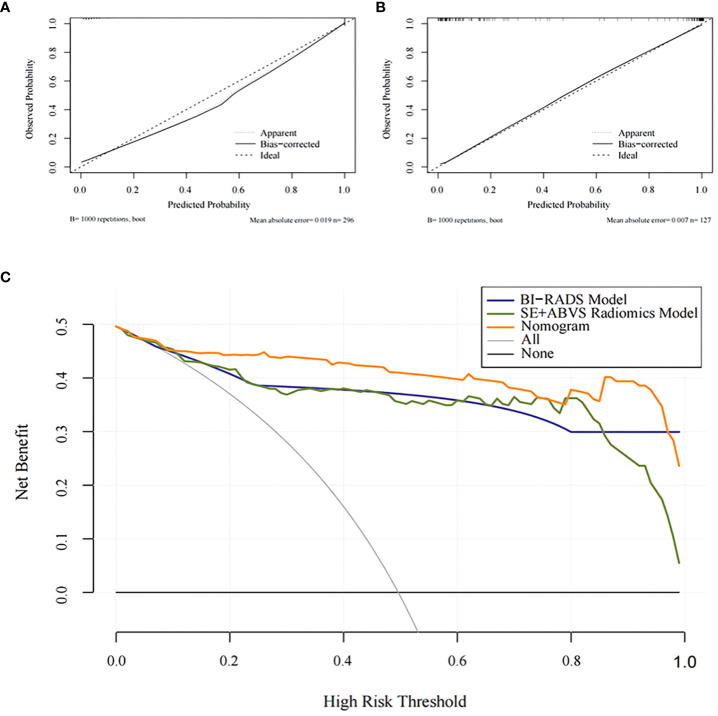
The calibration curves for the Nomogram in the training group **(A)** and the validation group **(B)**.The decision analysis curves of the BI-RADS mode, SE+ABVS radiomics model, and Nomogram in the validation group **(C)**.

The calibration curve exhibits a favorable fit of the nomogram (**Figures 7A, B**). indicating that the predicted risk by the nomogram was close to the observed risks. The results from the Hosmer-Lemeshow test further proved that the differences between them did not present statistical significance in either the training group (*P*=0.70) or validation group (*P*=0.95).

The clinical decision analysis curve (**Figure 7C**) indicates that utilizing the BI-RADS model, SE+ABVS radiomics model, and nomogram for decision-making significantly improved the net benefit for patients compared to the assumption of intervention for all lesions or no intervention at all. Furthermore, the nomogram provided a greater net benefit to patients compared to both the BI-RADS model and SE+ABVS radiomics model.

## 4 Discussion

The study combined radiomics features of ABVS and SE images with conventional imaging diagnosis criteria along with clinical risk factors for developing a clinical-radiomics nomogram that demonstrated excellent diagnostic efficacy, as well as good calibration capabilities, and significant clinical usefulness.

Although ABVS and SE examination techniques offer significant advantages in breast screening, the examiner’s naked eye remains incapable of capturing deep image information. Radiomics provides a pathway to capture internal tumor information at a more profound level. Wang et al. derived radiomics features from ABVS images and constructed multiple machine learning models for breast cancer diagnosis, the best of which was the support vector machine model with an AUC of 0.857 (17). Additionally, Liu et al. employed radiomics features extracted from SE images for breast cancer prediction, yielding a Radscore with an AUC of 0.866 in the test set (18). Besides, Ma et al. developed a multivariate logistic model by combining SE, B-mode, and ABVS coronal radiomics features, with an AUC value of 0.946 in the internal validation group (19). In this study, we performed radiomics analysis on both ABVS and SE images. For ABVS images, we delineated the ROI across sagittal, transverse, and coronal planes. While outlining the ROI in the ABVS coronal planes and the SE image, we incorporated a portion of the lesion’s peripheral tissues to capture additional information. As a result, the ABVS and SE features that we acquired demonstrated good predictive capabilities for breast cancer, and the combination of the two yielded a higher diagnostic efficacy than the BI-RADS model that obtained by a highly experienced physician based on visual assessment alone (AUC: 0.933 vs. 0.916). Additionally, this study analyzed clinical risk factors related to breast cancer and revealed that age and lesion size exhibited significantly higher values in the malignant group compared to the benign group, which is consistent with previous research findings (33–35). Therefore, we developed a nomogram by integrating Radscore, patient’s age, maximum diameter of the lesion, and BI-RADS scores using multivariate logistic regression analysis. The AUC of this nomogram in the internal validation group was 0.964, which surpassed that of both the SE+ABVS radiomics model and the clinical model. Furthermore, we conducted an analysis on the clinical utility of this nomogram, and the decision analysis curves revealed that it could offer superior net benefit to patients across a broad range of threshold intervals. Consequently, the nomogram holds significant value as a point of reference for clinicians, particularly novice practitioners lacking diagnostic expertise in identifying suspicious lesions.

In addition, this nomogram has demonstrated significant advantages in the diagnosis of BI-RADS category 4 lesions. The appearance of these lesions on imaging can be highly deceptive, so they span a wide range of malignancy risks (36, 37), which makes clinical diagnosis extremely challenging, often necessitating biopsies to definitively determine the nature of such lesions (30). However, routine biopsy results are often influenced by the spatial heterogeneity of the lesion and operator expertise (38), while also being an invasive procedure with potential complications such as bleeding (39). The majority of radiomics studies for this category of lesions have predominantly utilized MR images, and these studies have yielded favorable outcomes (26–29). Nevertheless, MR examinations are expensive, time-consuming, and not suitable for common screenings (40, 41). Based on ABVS images, Wang et al. integrated clinical ultrasound factors and Radscore to develop a nomogram for the diagnosis of BI-RADS category 4 lesions, which achieved an AUC value of 0.925 in the internal validation group and effectively minimized unnecessary biopsies (20). During this study, we constructed nomogram that also achieved an AUC value of 0.930 for the diagnosis of BI-RADS category 4 lesions in the validation group, surpassing the performance of the clinical model (AUC: 0.839), thereby further validating its good diagnostic efficacy. This may be attributed to the fact that the radiomics features selected for this study are all texture features with the majority derived from wavelet transform. Previous studies have demonstrated the value of wavelet transform-based texture features for the diagnosis of tumor lesions (42). The primary advantage of wavelet transform in image analysis lies in its multi-scale analysis capability, allowing it to capture the texture information of an image at various granularities. It possesses directional sensitivity, enabling it to accurately identify texture changes in multiple directions, while its time-frequency localization property allows it to keenly detect local variations in images. Additionally, wavelet transform can enhance image contrast, exhibit certain resistance to noise, and effectively compress image information, making feature extraction more robust and efficient (43). By quantifying the textural variances of breast lesions, we successfully captured the subtle heterogeneity within these lesions, thereby effectively distinguished between benign and malignant breast lesions.

Lambin et al. introduced the radiomics quality score (RQS) to provide a framework for clinical researchers to evaluate and guide their radiomics studies (44). This study has given comparatively detailed elaboration on image acquisition, feature extraction and screening, and model construction in order to ensure the reproducibility of the study. Two physicians independently delineated the lesions, effectively achieving multiple segmentations. The features extracted from both segmentations were then subjected to ICC analysis. Consequently, only the features demonstrating excellent repeatability and robustness were selected. To prevent model overfitting, we standardized the feature values. Features with strong discriminative ability were obtained through the U-test, LASSO regression with tenfold cross-validation from ABVS and SE images, respectively. Subsequently, we evaluated the constructed nomogram using calibration curves and the Hosmer-Lemeshow test, which demonstrated excellent calibration performance. Based on these analyses, the nomogram appears to be a robust and generalizable tool, offering accurate risk prediction with potential for practical clinical implementation. Although the development of this nomogram necessitates a combination of diverse factors, these data can be retrospectively obtained without imposing an additional examination burden on patients.

Admittedly, This study was subject to certain limitations: It was conducted as a single-center retrospectively study thus selection bias may have occurred, and lacked external validation, which necessitates further multicenter large-sample studies and prospective trials for the validation of our developed nomogram.

## 5 Conclusion

The nomogram developed in this study, which combined SE and ABVS radiomics features, with traditional imaging assessment criteria and clinical risk factors, it can serve as a reliable and non-invasive analytical tool to assist physicians in accurately assessing the malignancy risk in solid hypoechoic breast lesions, leading to better clinical decision-making.

## Data availability statement

The original contributions presented in the study are included in the article/supplementary material. Further inquiries can be directed to the corresponding author.

## Ethics statement

The studies involving humans were approved by Institutional Review Board of the Third Xiangya Hospital, CSU. The studies were conducted in accordance with the local legislation and institutional requirements. Written informed consent for participation was not required from the participants or the participants’ legal guardians/next of kin in accordance with the national legislation and institutional requirements.

## Author contributions

GS: Conceptualization, Formal Analysis, Methodology, Writing – original draft, Writing – review & editing. JL: Formal Analysis, Methodology, Writing – review & editing. YY: Data curation, Writing – review & editing. ZY: Conceptualization, Data curation, Formal Analysis, Funding acquisition, Supervision, Writing – review & editing.
